# Portable and Wearable Brain Technologies for Neuroenhancement and Neurorehabilitation

**DOI:** 10.1155/2018/1806374

**Published:** 2018-06-19

**Authors:** Noman Naseer, Hasan Ayaz, Frederic Dehais

**Affiliations:** ^1^Department of Mechatronics Engineering, Air University, Islamabad, Pakistan; ^2^Drexel University, School of Biomedical Engineering, Science and Health Systems, Philadelphia, PA, USA; ^3^University of Pennsylvania, Department of Family Medicine and Community Health, Philadelphia, PA, USA; ^4^Children's Hospital of Philadelphia, The Division of General Pediatrics, Philadelphia, PA, USA; ^5^Institut Supérieur de l'Aéronautique et de l'Espace, Université de Toulouse, France

The recent advent of portable and wearable neuroimaging and neurostimulation technologies triggered a proliferation of research on brain recording and augmentation, both in healthy adults and in patients with neurological or psychiatric disease. Augmentation refers to the improvement of brain function (e.g., cognitive, affective, and motor) through task performance or reversal of deficits that are normal consequences of performance in healthy adults (e.g., mental fatigue and stress) or those related to brain disorders. The main objective of this special issue was to bring together recent advances in clinical and field applications of portable and wearable brain technologies, like neuroimaging, such as electroencephalography (EEG), functional near-infrared spectroscopy (fNIRS), and also neurostimulation approaches like transcranial direct-current stimulation (tDCS). Such approaches have made significant progress in recording and altering brain activity while allowing full body movements outside laboratory environments.

This special issue contains eight published works (six original research articles and two review articles) selected from 17 submitted articles addressing novel trends of various portable and wearable brain technologies that can be used for neuroenhancement and neurorehabilitation. O. Dehzangi and M. Farooq present the design of a portable brain-computer interface (BCI) system that is optimized to operate effectively in intensive care unit environment. Their system consists of a wearable EEG cap together with an Android app designed on a mobile device that serves as visual stimuli and data processing module. Furthermore, in order to overcome the challenges that BCI systems face nowadays in real-world scenarios, they propose a novel subject-specific Gaussian Mixture Model (GMM) based training and adaptation algorithm. F. Cieri and R. Esposito review the state-of-the-art resting-state functional magnetic resonance (fMRI) studies about physiology and pathology of aging process and suggest future direction in this field of research and its potential clinical applications. U. Ghani et al. propose a method that can facilitate online processing of EEG signals by providing an efficient filtering implementation in a hardware friendly environment by switching to finite impulse response (FIR). They minimize latency and computational delay of preprocessing related to any EEG based BCI applications. A. D. Bateson et al. present an interesting scheme, Categorization of Mobile EEG (CoME), to enable researchers to quantify the degree of device mobility, participant mobility, and system specification used in their mobile EEG investigations in a standardized way. S. Blum et al. present the development and validation of a modular signal processing and classification application enabling online electroencephalography (EEG) signal processing on off-the-shelf mobile Android devices. The software application SCALA (Signal ProCessing and CLassification on Android) supports a standardized communication interface to exchange information with external software and hardware. T. Kondo et al. evaluate the difference between the therapeutic effect of low-frequency repetitive transcranial magnetic stimulation (LF-rTMS) and that of continuous theta burst stimulation (cTBS), when each is combined with intensive occupational therapy (OT), in poststroke patients with upper limb hemiparesis. They recommend the use of 2400 pulses of LF-rTMS/OT for 2 weeks as treatment for hemiparetic patients. I. Fajnerova et al. present a resting-state fMRI study to conclude that externalization of spatial navigation to technological device (GPS in AR glasses) can decrease the functional coupling between hippocampus and associated brain regions. N. Rashid et al. present a design of embedded system for multivariate classification of finger and thumb movements using EEG signals for control of upper limb prosthesis. They conclude that a two-stage logistic regression classifier exhibits highest classification accuracy when power spectral density is extracted as feature from filtered EEG signals.

In summary, the papers in this series highlight several important research strategies that are making it increasingly evident that the neuroimaging findings and neurostimulation interventions are of translational value for concurrent real-world challenges. The results from these brain technology studies not only help us to understand the brain processes in complex realistic tasks but also show great potential to provide urgently needed objective biomarkers for clinical diagnosis, evaluation, and therapy.

